# MELD-S prognostic model: development and external validation for 90-day mortality prediction in acute-on-chronic liver failure

**DOI:** 10.3389/fimmu.2026.1881613

**Published:** 2026-10-21

**Authors:** Zeqiang Qi, Tingyu Guo, Chang Liu, Xing Zhang, Mo Zhang, Ying Xiao, Luyuan Ma, Yadong Wang

**Affiliations:** 1 Department of Infectious Diseases, Hebei Medical University Third Hospital, Shijiazhuang, China; 2 Department of Gastroenterology, Dingzhou People’s Hospital of Hebei Province, Dingzhou, China

**Keywords:** acute-on-chronic liver failure, FOXO1 pathway, model for end-stage liver disease, prognostic prediction, serum/glucocorticoid-regulated kinase 1

## Abstract

**Background and objectives:**

Accurate prognostic assessment of acute-on-chronic liver failure (ACLF) remains a major clinical challenge. Existing models, such as the Model for End-Stage Liver Disease (MELD), rely solely on clinical indicators and do not incorporate molecular biomarkers potentially associated with hepatic regenerative processes. This study aimed to identify key regeneration-associated genes through bioinformatics, integrate the most promising biomarker with clinical parameters to construct a novel 90-day mortality prediction model, and validate its performance for improving risk stratification in ACLF.

**Methods:**

Transcriptomic data from an ACLF cohort (GSE139602) and a liver regeneration dataset (GSE15239) were obtained from the Gene Expression Omnibus (GEO) database. Differentially expressed genes (DEGs) were screened, followed by functional enrichment analysis and protein-protein interaction (PPI) network construction. From August 2023 to July 2024, 64 patients with ACLF were prospectively enrolled and categorized based on 90-day outcomes. After 1:1 propensity score matching for age, sex, MELD score, and complications, 50 patients (25 survivors, 25 non-survivors) were selected as the development cohort. Separately, from August 2024 to July 2025, 52 patients with ACLF were enrolled, and after identical matching, 40 patients (20 survivors, 20 non-survivors) formed the independent external validation cohort. The matched development cohort was used for exploratory model development, while the independent external validation cohort was used to evaluate reproducibility. Serum levels of proteins encoded by core genes (SGK1, GADD45β, FOXO1) were measured using enzyme-linked immunosorbent assay (ELISA). The MELD-S model, integrating MELD score and SGK1, was developed via multivariate logistic regression. Its performance was evaluated through ROC analysis, calibration curves, Hosmer-Lemeshow test, and decision curve analysis (DCA) in both the development and external validation cohorts, with a nomogram created for visualization.

**Results:**

Bioinformatics analysis identified 20 key genes commonly dysregulated in ACLF and liver regeneration, enriched in transcription-related functions and the FOXO signaling pathway. SGK1, GADD45β, and FOXO1 were identified as candidate regeneration-associated genes, with significant protein interactions. In the development cohort, serum levels of SGK1, GADD45β, FOXO1, PT, and INR were significantly higher in non-survivors compared with survivors (all *P* < 0.05). Serum SGK1 levels showed a positive correlation with MELD score (r = 0.316, *P* = 0.027) and SGK1 was identified as an independent risk factor for 90-day mortality (OR = 3.955, 95% CI: 1.486 – 10.525, *P* = 0.006). The integrated MELD-S model yielded an AUC of 0.865 (95% CI: 0.767–0.963), demonstrating superior discriminative ability to MELD alone (AUC = 0.735) or SGK1 alone (AUC = 0.832). In the independent external validation cohort, the MELD-S model achieved an AUC of 0.725 (95% CI: 0.563–0.887, *P* = 0.015), compared with an apparent AUC of 0.865 in the development-cohort. Bootstrap internal validation of the development cohort yielded an optimism-adjusted AUC of 0.842.

**Conclusion:**

SGK1 is an independent prognostic biomarker associated with disease severity and biological stress responses in ACLF. The MELD-S model shows promising prognostic performance and may have potential clinical utility, offering a practical tool for risk assessment and individualized risk stratification. The biological mechanisms linking SGK1 to ACLF outcomes, including the potential involvement of the SGK1–FOXO1 pathway, remain hypothetical and require direct mechanistic validation in future studies.

## 1 Introduction

Acute-on-chronic liver failure (ACLF) is a severe clinical syndrome characterized by acute decompensation in patients with underlying chronic liver disease triggered by insults such as infection, alcohol, or drugs. Marked by high rates of multiple organ failure and substantial short-term mortality, it imposes a substantial burden on global healthcare systems (1, 2). Recent epidemiological studies reveal that ACLF occurs in approximately 35% of patients admitted with decompensated cirrhosis worldwide, with the highest prevalence of 65% reported in South Asia (2), with HBV infection, alcohol-associated liver disease, and other etiologies contributing to ACLF development across different regions. Recently proposed diagnostic frameworks, including the China-CLIF criteria, emphasize standardized identification and risk stratification of ACLF across diverse etiological backgrounds. A recent multicohort study evaluated this framework across multiple etiologies and geographic regions, supporting its broader applicability for ACLF classification and prognostic assessment (3). Accurate prognostic assessment is vital for clinical decision-making and resource allocation, where the 90-day timepoint is widely adopted as a critical determinant for evaluating long-term outcomes (4). Currently, prognostic models such as the Model for End-Stage Liver Disease (MELD) and the EASL-CLIF Consortium score are widely used in clinical practice. These systems effectively reflect the degree of hepatic dysfunction and systemic injury based on routine clinical and laboratory parameters (4). Established ACLF-specific prognostic scores, including the CLIF-C ACLF, AARC, and COSSH-ACLF scores, are also used for severity assessment and prognostic evaluation. However, they primarily capture the extent of liver injury but generally do not include molecular biomarkers that may provide additional information regarding hepatic regenerative responses, a key determinant of recovery and survival in ACLF (5). Accumulating evidence indicates that impaired hepatocyte regeneration is a central pathological feature driving poor outcomes in ACLF. This highlights a significant limitation of existing prognostic tools, which capture the extent of injury but not the capacity for recovery. Therefore, constructing a prognostic model that integrates functional molecular markers of hepatocyte regeneration is crucial for identifying long-term outcomes in patients with ACLF.

In clinical practice, ACLF prognosis is influenced by the type of underlying liver disease, complications, and the number and severity of involved organs. However, impaired hepatocellular regenerative capacity is also a key factor in adverse outcomes. Previous studies have confirmed that hepatocellular regeneration in ACLF is profoundly suppressed. Key signaling pathways, including serum/glucocorticoid-regulated kinase 1 (SGK1)- and FOXO1-mediated signaling and Wnt/β-catenin pathway, are significantly regulated, thereby compromising cell self-repair mechanisms (6–8). For instance, within the inflammatory milieu of ACLF, cytokines like TNF-α and IL-6 lead to cytoplasmic sequestration of FOXO1 by activating the PI3K/Akt pathway, subsequently inhibiting pro-regenerative gene transcription and hepatocyte proliferation (9). Despite these insights, clinically applicable biomarkers that quantitatively reflect regenerative potential remain elusive. The integration of bioinformatics approaches with transcriptomic data from public repositories like the Gene Expression Omnibus (GEO) offers a promising strategy to identify core genes associated with liver regeneration in ACLF, bridging molecular mechanisms with clinical phenotypes (10). In addition, validating the expression patterns of these key genes in ACLF patients is important for diagnosis, treatment, and prognostic assessment of ACLF.

Therapeutic strategies for ACLF remain limited and primarily supportive. Orthotopic liver transplantation is the only curative intervention, but its application is constrained by donor organ scarcity and high costs. Alternative strategies, such as stem cell therapy, which aims to promote hepatocyte regeneration and mitigate apoptosis and inflammatory necrosis, hold potential but require further validation of their long-term safety and efficacy (11). Similarly, artificial liver support systems serve as a crucial adjunct to internal medicine therapy, temporarily ameliorating the internal milieu by removing toxins and inflammatory mediators. However, their ultimate success is contingent upon the intrinsic regenerative capacity of the residual hepatocytes (12). These inherent therapeutic limitations underscore the urgent need for refined prognostic models capable of identifying patient subgroups most likely to derive benefit from specific interventions.

To address this unmet need, the present study integrated bioinformatics discovery with prospective clinical validation to identify candidate liver regeneration-associated genes and evaluate their potential prognostic relevance and develop a clinically applicable prognostic tool for ACLF. We quantified serum levels of proteins encoded by these genes in a prospectively enrolled ACLF cohort and analyzed their correlations with clinical features and outcomes. Furthermore, we developed and validated a novel prognostic model, named MELD-S (integrating the SGK1 and MELD score). This integrated approach aims to provide a biomarker-integrated tool for predicting 90-day prognosis, thereby supporting personalized clinical management in ACLF.

## 2 Materials and methods

### 2.1 Bioinformatics analysis for identification of liver regeneration-related genes

#### 2.1.1 Data acquisition

We obtained two publicly available human liver-tissue expression datasets from the Gene Expression Omnibus (GEO) database. GSE139602 included liver-biopsy transcriptomes from 8 patients with ACLF and 6 healthy controls and was generated using the Affymetrix Human Genome U219 array. GSE15239 included human liver samples collected after partial hepatectomy and healthy liver controls and was generated using the Affymetrix Human Genome U133 Plus 2.0 array.

#### 2.1.2 Screening of differentially expressed genes

DEGs were identified for each dataset separately using the “limma” package (version 3.50.0) in R (version 4.4.3). Genes with an absolute log2 fold change (|log2FC|) > 1 and an adjusted *P*-value (false discovery rate, FDR) < 0.05 were considered statistically significant. The overlapping DEGs common to both ACLF and liver regeneration datasets were identified as liver regeneration-related candidate genes, and visualized using a Venn diagram. Each dataset was analyzed separately, and expression values were not pooled across datasets. Only genes showing the prespecified opposite direction of change (downregulated in ACLF and upregulated in the liver regeneration dataset) in the two analyses were intersected. Therefore, the overlapping genes were used to generate candidates rather than to define a directly validated molecular signature.

#### 2.1.3 Functional enrichment analysis

Gene Ontology (GO) and Kyoto Encyclopedia of Genes and Genomes (KEGG) pathway enrichment analyses were conducted for the overlapping DEGs using the clusterProfiler package (version 4.0.5) in R software. GO analysis covered Biological Process (BP), Cellular Component (CC), and Molecular Function (MF). The corresponding proteins encoded by the core genes were mapped using the UniProt database (https://www.uniprot.org/). KEGG analysis identified significantly enriched signaling pathways.

#### 2.1.4 Protein-protein interaction network construction

A PPI network for the core proteins was constructed using the STRING database (version 11.5, https://string-db.org/) with a minimum required interaction confidence score >0.7. The resulting network was imported into Cytoscape software (version 3.9.1) for visualization and further analysis. Node degree was defined as the number of direct interaction edges connected to each protein node and was calculated using the NetworkAnalyzer tool in Cytoscape. Candidate hub proteins were selected according to their numerically calculated node-degree values rather than their visual position in the network layout.

### 2.2 Study population and clinical validation

#### 2.2.1 Patient enrollment and ethical considerations

Between August 2023 and July 2024, 64 consecutive patients diagnosed with ACLF at Hebei Medical University Third Hospital were prospectively enrolled. Based on 90-day outcomes, they were categorized into survivors (n=34) and non-survivors (n=30). To reduce baseline imbalance between survivors and non-survivors in this exploratory cohort with limited sample size, 1:1 propensity score matching (PSM) based on age, sex, MELD score, and major complications was performed, yielding a matched development cohort of 50 patients (25 survivors, 25 non-survivors). Separately, an independent cohort of 52 patients with ACLF enrolled between August 2024 and July 2025 underwent identical PSM, resulting in an external validation cohort of 40 patients (20 survivors, 20 non-survivors). Because PSM may alter the original clinical distribution, sensitivity analyses were additionally performed using the complete unmatched development cohort (n=64) and external validation cohort (n=52). The final MELD-S equation derived from the matched development cohort was applied without refitting to evaluate whether the observed discrimination remained consistent in the original populations.

The sample size was estimated *a priori* using SPSS based on a pilot study. Assuming a mean difference in serum SGK1 of 1.54 ng/mL between the survivor and non-survivor groups, a standard deviation of 2.12 ng/mL, a two-sided α of 0.05, and a power (1-β) of 0.8, the minimum required sample size was 48. The study protocol was approved by the Hospital’s Medical Ethics Committee (Approval No: 2022-118-1). Written informed consent was obtained from all patients or their legal guardians prior to enrollment, and the study was conducted in accordance with the ethical principles of the Declaration of Helsinki.

#### 2.2.2 Diagnostic criteria

ACLF was diagnosed according to the 2019 guidelines of the Asian Pacific Association for the Study of the Liver (APASL) (13). The diagnosis was established based on the following criteria: pre-existing chronic liver disease (e.g., chronic hepatitis B/C, liver cirrhosis, alcoholic liver disease) ≥ 6 months, presentation with jaundice (total bilirubin ≥ 5 mg/dL) and coagulopathy (international normalized ratio ≥ 1.5), complicated by ascites and/or hepatic encephalopathy within 4 weeks. Disease severity was graded using the APASL ACLF Research Consortium (AARC) score (14).

Inclusion criteria were: (1) Age between 18 and 75 years, regardless of gender or etiology; (2) Meeting the APASL diagnostic criteria for ACLF.

Exclusion criteria were: (1) severe pre-existing cardiac dysfunction (NYHA class III–IV), pre-existing chronic renal dysfunction defined as a serum creatinine concentration >451 μmol/L before ACLF onset, or pre-existing chronic respiratory failure requiring mechanical ventilation before study enrollment; (2) history of liver transplantation or stem cell therapy within the preceding 3 months; (3) psychiatric or cognitive disorders impairing follow-up compliance; (4) pregnancy or lactation; or (5) concurrent diagnosis of any malignancy. These criteria were intended to reduce confounding from irreversible pre-existing organ dysfunction. Acute renal or respiratory organ failure developing after ACLF onset did not constitute an exclusion criterion and was considered part of the clinical course of ACLF.

### 2.3 Data collection and laboratory measurements

#### 2.3.1 Baseline clinical data

Demographic and clinical data were collected from the hospital’s electronic medical record system at admission, including age, gender, smoking and alcohol history, etiology of the underlying liver disease, comorbidities (e.g., hypertension, diabetes), and complications at presentation (e.g., hepatic encephalopathy, infection, ascites, renal insufficiency, esophagogastric variceal bleeding).

#### 2.3.2 Treatment, follow-up, and grouping

All patients received standardized medical management per prevailing clinical guidelines. Complications arising during hospitalization, including infections, bleeding events, and progression of hepatic encephalopathy or renal insufficiency, were meticulously recorded. Patients were followed for 90 days after discharge via telephone, WeChat, or outpatient visits. Based on 90-day outcome, they were categorized into the survivor and non-survivor group; the latter included patients who underwent liver transplantation within 90 days.

#### 2.3.3 Routine laboratory tests

Venous blood samples were collected at admission for routine laboratory analysis. Hematological parameters: white blood cell (WBC), neutrophil (NEUT), lymphocyte (LYM), red blood cell (RBC), and platelet (PLT) counts were determined using an automated hematology analyzer; Biochemical parameters: Serum levels of albumin (ALB), alanine aminotransferase (ALT), aspartate aminotransferase (AST), total bilirubin (TBil), direct bilirubin (DBil), alkaline phosphatase (ALP), γ-glutamyl transferase (GGT), sodium (Na^+^), and creatinine (Scr) were measured using an automated biochemical analyzer; Coagulation profile: prothrombin time (PT), activated partial thromboplastin time (APTT), fibrinogen (Fib), and D-dimer were measured using an automated coagulation analyzer. Prothrombin activity (PTA) and the international normalized ratio (INR) were calculated; Serum alpha-fetoprotein (AFP) levels were quantified using a chemiluminescent immunoassay analyzer; The Model for End-Stage Liver Disease (MELD) score was calculated as: 3.78 × ln[TBil (mg/dL)] + 11.2 × ln[INR] + 9.57 × ln[Scr (mg/dL)] + 6.43×etiology (0 for biliary or alcoholic, 1 for others).

#### 2.3.4 Measurement of molecular indicators

Fasting peripheral venous blood (5 mL) was collected at enrollment. Serum was separated by centrifugation at 3000 rpm for 10 minutes and stored at -80 °C until analysis. Serum concentrations of SGK1, growth arrest and DNA-damage-inducible protein 45 beta (GADD45β), and FOXO1 were quantified using commercial enzyme-linked immunosorbent assay (ELISA) kits (Jingmei Biotechnology Co., Ltd., China) according to the manufacturer’s protocols. The intra- and inter-assay coefficients of variation were both <15%, and the minimum detectable concentration for all assays was <1.0 pg/mL.

### 2.4 Statistical analysis

Statistical analyses were performed using SPSS Statistics (version 27.0) and R software (version 4.4.0). Continuous variables with normal distribution were presented as mean ± standard deviation ( $\bar{X}$ ± SD) and compared using independent samples t-test. Non-normally distributed data were presented as median (interquartile range) [M (P25, P75)] and compared using Mann-Whitney U test. Categorical variables were expressed as frequencies or percentages (%) and compared using Chi-square (χ²) test. Correlations between molecular indicator levels and MELD scores were assessed using Spearman’s rank correlation coefficient.

Univariate logistic regression was employed to identify potential factors associated with 90-day mortality. Candidate variables with a univariable *P* value < 0.10 were considered for multivariable logistic regression. Because MELD score was collinear with INR, the first multivariable model excluded MELD score and used backward stepwise selection (based on the Akaike information criterion) among the remaining candidate variables. After SGK1 was retained as an independent predictor, a second multivariable model including SGK1 and MELD score, but not INR, was fitted to estimate the final MELD-S coefficients.

The performance of the MELD-S model was evaluated by the area under the receiver operating characteristic (ROC) curve (AUC) with 95% confidence intervals. Calibration of the model was assessed using the Hosmer-Lemeshow test and calibration curves. Clinical utility was evaluated via decision curve analysis (DCA). To assess potential optimism caused by model development in a limited sample, bootstrap resampling with 1, 000 repetitions was performed for internal validation, and optimism-adjusted performance estimates were calculated. Finally, a nomogram was developed for visualization of individualized mortality risk prediction.

The performance of the finalized MELD-S model was subsequently assessed in the independent external validation cohort. As a sensitivity analysis, the predictive performance of MELD-S and MELD was additionally evaluated in the complete unmatched cohorts. The previously established MELD-S equation was applied directly without model recalibration or refitting. The area under the ROC curve (AUC) with 95% confidence intervals was calculated, and differences between MELD-S and MELD were compared using DeLong’s test.

In addition to the MELD-S model, established ACLF prognostic scores, including the APASL ACLF Research Consortium (AARC) score, CLIF-C ACLF score, and COSSH-ACLF score, were calculated based on baseline clinical parameters. The predictive performance of these models for 90-day mortality was compared using ROC curve analysis, and differences between ROC curves were assessed using DeLong’s test.

A two-tailed *P*-value < 0.05 was considered statistically significant.

### 2.5 Prediction model development and reporting guidelines

The development, validation, and reporting of the prognostic prediction model strictly adhered to the Transparent Reporting of a multivariable prediction model for Individual Prognosis or Diagnosis (TRIPOD) Statement (15). According to the TRIPOD statement, this study involved model development using a prospective clinical cohort followed by evaluation in an independent external validation cohort. Therefore, the study design corresponds to development and external validation of a prediction model (TRIPOD Type 3). A completed TRIPOD+AI checklist is provided as **Supplementary Material**.

## 3 Results

### 3.1 Identification of hepatocyte regeneration-associated core genes

#### 3.1.1 Screening of differentially expressed genes

Using a threshold of |log2FC| > 1 and FDR < 0.05, comparative transcriptomic analysis of the GSE139602 dataset identified 1, 570 DEGs between patients with ACLF and healthy controls, comprising 793 upregulated and 777 downregulated genes (**Figure 1a**). In the liver regeneration following partial hepatectomy dataset GSE15239, 277 DEGs were identified, including 204 upregulated and 73 downregulated genes (**Figure 1b**). Intersection analysis identified 20 genes that were downregulated in ACLF and upregulated during liver regeneration. These included PHLDA1, ADM, APOLD1, GADD45β, CYTIP, NFIL3, TIPARP, PNRC1, SGK1, KLHL15, CREM, C9orf72, DDX21, FCGR3B, FOXO1, NR5A2, RGS2, SAMSN1, ZFAND5, and OSBPL11 (**Figure 1c**). Given the limited sample sizes of these public datasets, the overlapping genes SGK1, GADD45β, and FOXO1 were prioritized as candidate regeneration-associated genes for subsequent clinical evaluation.

**Figure 1:**
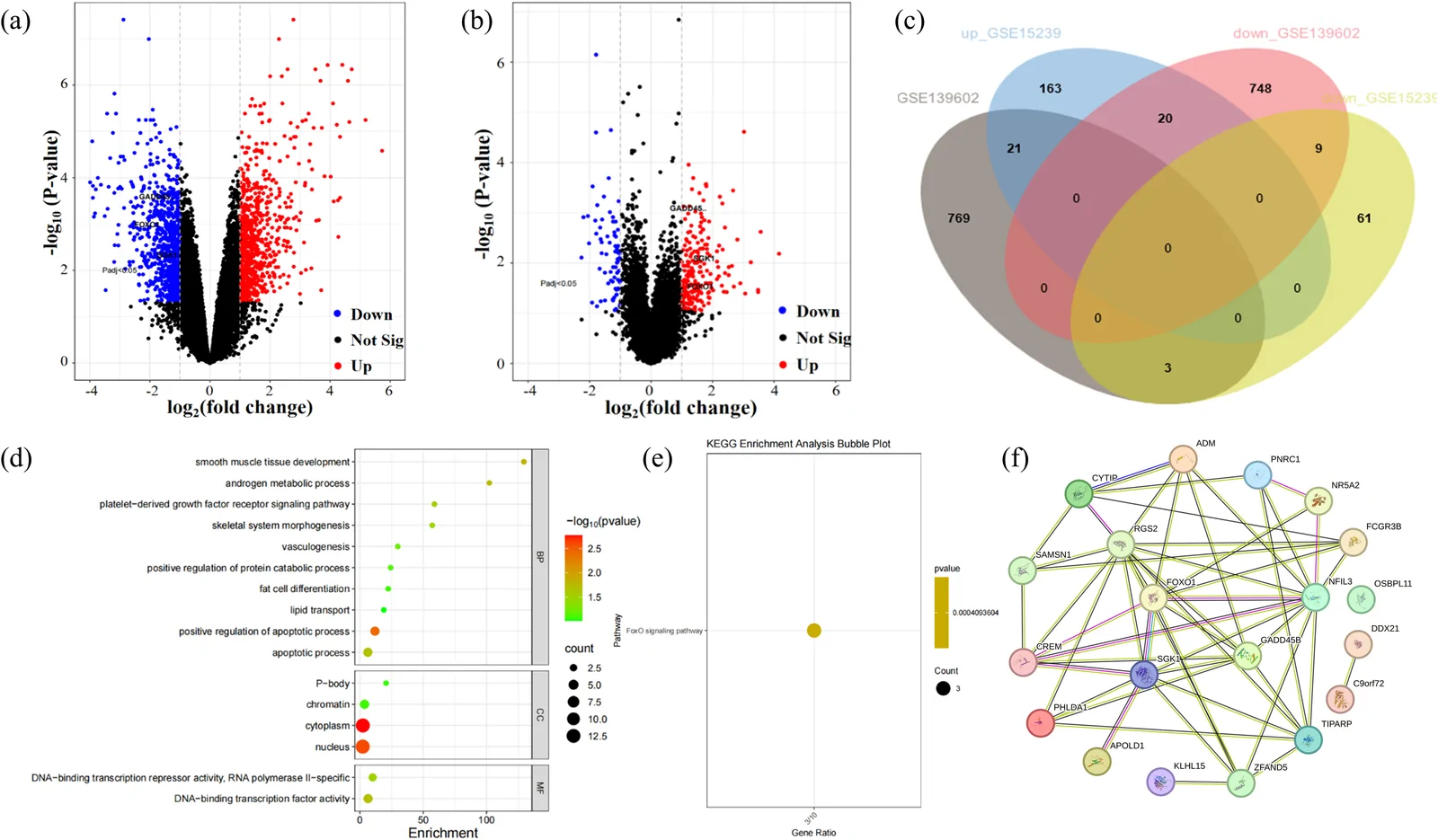
Identification and functional characterization of hepatocyte regeneration-related genes in ACLF. **(a)** Volcano plot of differentially expressed genes (DEGs) between ACLF patients and healthy controls (GSE139602). Genes with |log_2_FC| > 1 and adjusted P < 0.05 were considered significant (red: up-regulated; blue: down-regulated; gray: non-significant). **(b)** Volcano plot of DEGs associated with liver regeneration after partial hepatectomy (GSE15239), analyzed using identical criteria. **(c)** Venn diagram identifying 20 overlapping DEGs consistently down-regulated in ACLF and up-regulated during hepatocyte regeneration. **(d)** Gene Ontology (GO) enrichment analysis of the 20 core genes. Bubble plot shows significantly enriched terms in biological process (BP), cellular component (CC), and molecular function (MF) categories; bubble size indicates gene count, color intensity represents –log_10_ (adjusted *P*-value). **(e)** Kyoto Encyclopedia of Genes and Genomes (KEGG) pathway enrichment analysis, highlighting the FOXO signaling pathway as the most significantly enriched. **(f)** Protein–protein interaction (PPI) network of proteins encoded by the core genes. SGK1, FOXO1, and GADD45β showed high connectivity within the PPI network (18, 16, and 14, respectively).

#### 3.1.2 Functional enrichment analysis by GO and KEGG

GO enrichment analysis indicated that the 20 core genes are involved in biological processes such as hormone metabolism, platelet-derived growth factor receptor signaling, angiogenesis, protein catabolism, lipid transport and apoptosis regulation. They were localized in cellular components including P-bodies (RNA metabolic granule formation), chromatin, cytoplasm, and nucleus, and were enriched in molecular functions related to specific activity of RNA polymerase II and DNA-binding transcription factor (**Figure 1d**). KEGG pathway analysis revealed significant enrichment for the FOXO signaling pathway (**Figure 1e**).

#### 3.1.3 Protein–protein interaction network analysis

PPI network analysis of the corresponding proteins revealed 112 interaction edges among 20 nodes. Based on the predefined node-degree calculation, SGK1, FOXO1, and GADD45β had degree values of 18, 16, and 14, respectively. These values were the highest among the 20 nodes, and the three proteins were therefore selected as candidate hub proteins. Although NFIL3 appeared visually central in the network layout, its calculated node degree was lower than that of the top three proteins. Visual centrality was not used as the criterion for hub-gene selection (**Figure 1f**).

### 3.2 Clinical characteristics of the ACLF cohort

#### 3.2.1 Baseline characteristics of patients with ACLF

In the development cohort, 25 survivors and 25 non-survivors based on PSM were selected from an initial pool of 64 individuals, and were included for model construction. The survivor group had a mean age of 53 ± 13 years (range 33–73 years), compared to 52 ± 9 years (range 33–70) in the non-survivor group. No significant differences were observed in gender, age, smoking or alcohol history, diabetes, liver disease etiology, or baseline complications including esophagogastric varices, hepatic encephalopathy, infection, ascites, and renal insufficiency (all *P* > 0.05). However, hypertension was more prevalent in the non-survivor group (24% vs. 4%, *χ*² = 4.153, *P* = 0.042) (**Supplementary Table 1**).

Similarly, the external validation cohort consisted of 40 patients (20 survivors and 20 non-survivors) derived from 52 ACLF cases after identical PSM. The survivor group had a mean age of 50 ± 9 years (range 33-71), whereas the non-survivor group had a mean age of 49 ± 12 years (range 29-74). No statistically significant differences were observed between the two groups in gender, age, etiology, or baseline complications (all *P* > 0.05) (**Supplementary Table 2**).

#### 3.2.2 Laboratory parameters

In the development cohort, non-survivors had significantly prolonged prothrombin time (PT) and international normalized ratio (INR), elevated total bilirubin levels, and higher MELD scores compared to survivors. Specifically, prolonged PT and INR, decreased PTA, increased TBil, and elevated MELD scores were key findings (all *P* < 0.05). Serum levels of SGK1, GADD45β, and FOXO1 were also significantly higher in non-survivors (SGK1: 1.99 *vs.* 0.41 ng/mL; GADD45β: 1.09 *vs.* 0.89 ng/mL; FOXO1: 1.70 *vs.* 1.32 ng/mL; Z = 3.980, 3.150, 3.469, all *P* < 0.001). No other laboratory parameters showed significant differences between the two groups (*P* > 0.05) (**Supplementary Table 3**).

In the external validation cohort, non-survivors similarly showed higher INR, lower PTA, higher MELD scores. Serum SGK1 level was also significantly higher in the non-survivors than survivors (1.19 vs. 0.80 ng/mL, Z=-3.381, *P* = 0.001). No other laboratory parameters showed significant differences between the two groups (*P* > 0.05) (**Supplementary Table 4**).

### 3.3 Prognosis analysis and construction of the MELD-S model

#### 3.3.1 Correlation analysis

Correlations between MELD score and serum levels of SGK1, GADD45β, FOXO1 were analyzed in all patients with ACLF. SGK1 and FOXO1 levels showed a positive correlation with MELD score (r = 0.316 and 0.322, respectively, both *P* = 0.027), while GADD45β showed no significant correlation with MELD score (r = 0.212, *P* = 0.153).

#### 3.3.2 Univariate and multivariate regression

Univariate logistic regression was performed with 90-day outcome as the dependent variable. When etiology was coded as a binary variable (viral vs. nonviral), viral etiology was associated with 90-day mortality in the univariate analysis (OR = 4.125, 95% CI: 1.092–15.585, *P* = 0.037) (**Table 1**). This analysis used a binary etiological variable, whereas the baseline comparison evaluated the overall distribution of etiological categories between survivors and non-survivors. Therefore, the two analyses addressed different hypotheses and their *P* values are not directly comparable. Viral etiology was not retained as an independent predictor in the final multivariable MELD-S model.

**Table 1:** Univariate logistic regression analysis for 90-day mortality.

Variable	Beta	SE	Statistics	*P* value	OR (95% CI)
Gender	-0.334	0.580	0.332	0.564	0.716 (0.230, 2.230)
Age	-0.006	0.029	0.044	0.833	0.994 (0.944, 1.047)
Weight	0.014	0.023	0.388	0.534	1.015 (0.969, 1.062)
Smoking history	0	0.570	0	1.000	1 (0.327, 3.055)
Alcohol history	-0.486	0.572	0.721	0.396	0.615 (0.201, 1.887)
Etiology (Virus)	1.417	0.678	4.366	0.037	4.125 (1.092, 15.585)
Comorbidity					
Diabetes	0	0.870	0	1.000	1 (0.182, 5.507)
Hypertension	2.025	1.123	3.253	0.071	7.579 (0.839, 68.461)
Complications at baseline					
Ascites	0.747	0.621	1.447	0.229	2.111 (0.625, 7.134)
HE	-0.736	1.259	0.341	0.559	0.479 (0.041, 5.652)
Infection	0.369	0.610	0.366	0.545	1.446 (0.438, 4.781)
Renal dysfunction	0.450	0.960	0.219	0.639	1.568 (0.239, 10.300)
New complications in hospitalization					
HE	0.904	0.694	1.699	0.192	2.471 (0.634, 9.625)
Infection	0	0.606	0	1.000	1 (0.305, 3.282)
EVB	1.415	1.074	1.735	0.188	4.117 (0.501, 33.804)
Renal dysfunction	21.531	15191	0	0.999	
Laboratory parameters at baseline					
WBC	-0.008	0.032	0.069	0.792	0.992 (0.931, 1.056)
NEUT	-0.003	0.036	0.006	0.940	0.997 (0.929, 1.070)
LYM	-0.417	0.355	1.378	0.24	0.659 (0.329, 1.322)
RBC	-0.270	0.336	0.644	0.422	0.763 (0.395, 1.476)
PLT	-0.003	0.004	0.680	0.410	0.997 (0.989, 1.004)
PT	0.08	0.034	5.506	0.019	1.084 (1.013, 1.159)
PTA	-0.029	0.015	3.555	0.059	0.972 (0.943, 1.001)
INR	0.914	0.388	5.554	0.018	2.493 (1.166, 5.329)
APTT	0.024	0.019	1.465	0.226	1.024 (0.986, 1.064)
Fib	-0.077	0.405	0.036	0.850	0.926 (0.419, 2.047)
D-dimer	0.070	0.060	1.404	0.236	1.073 (0.955, 1.206)
ALB	-0.006	0.073	0.008	0.931	0.994 (0.862, 1.146)
ALT	-0.002	0.001	1.700	0.192	0.998 (0.996, 1.001)
AST	-0.003	0.002	2.284	0.131	0.997 (0.994, 1.001)
TBiL	0.005	0.003	3.169	0.075	1.005 (0.999, 1.011)
ALP	0.002	0.002	0.932	0.334	1.002 (0.998, 1.007)
GGT	0	0.001	0.026	0.871	1.000 (0.998, 1.002)
Na+	0.033	0.046	0.516	0.472	1.034 (0.944, 1.132)
SCr	0.008	0.005	1.999	0.157	1.008 (0.997, 1.018)
MELD	0.150	0.056	7.306	0.007	1.162 (1.042, 1.295)
MELD-Na	0.040	0.026	2.309	0.129	1.041 (0.989, 1.096)
SGK1	1.312	0.477	7.563	0.006	3.713 (1.458, 9.458)
GADD45β	0.359	0.153	5.543	0.019	1.432 (1.062, 1.931)
FOXO1	0.380	0.175	4.744	0.029	1.462 (1.039, 2.059)

HE, hepatic encephalopathy; EVB, esophagogastric variceal bleeding; WBC, white blood cell; NEUT, neutrophilic granulocyte; LYM, lymphocyte; RBC, red blood cell; PLT, platelets; PT, prothrombin time; PTA, prothrombin activity; INR, international normalized ratio; APTT, activated partial thromboplastin time; Fib, fibrinogen; ALB, albumin; ALT, alanine aminotransferase; AST, aspartate aminotransferase; TBil, total bilirubin; ALP, alkaline phosphatase; GGT, gamma-glutamyl transpeptidase; Na, sodium; SCr, serum creatinine; MELD, model for end stage liver disease; SGK1, serum/glucocorticoid-regulated kinase 1; GADD45β, growth arrest and DNA-damage-inducible protein 45 beta; FOXO1, forkhead box protein O1. Etiology was analyzed as viral versus nonviral in the logistic regression model.

Because MELD score was collinear with INR, the first multivariable logistic regression model excluded MELD score and used backward stepwise selection among the remaining candidate variables. In this first model, only SGK1 remained an independent predictor of 90-day mortality (OR = 3.955, 95% CI: 1.486–10.525, *P* = 0.006). After SGK1 was identified as an independent predictor in the first model, a second multivariable logistic regression model including SGK1 and MELD score, but excluding INR, was fitted to estimate the coefficients used to construct the final MELD-S equation: Logit(P) = –6.892 + 0.132 × MELD + 1.442 × SGK1 (**Table 2**). Thus, the MELD coefficient was estimated in the second model that simultaneously included MELD score and SGK1; it was not derived from the first model in which MELD score had been excluded.

**Table 2:** Multivariate logistic regression analysis for the MELD-S model.

Variable	Beta	SE	Statistics	*P* value	OR (95% CI)
MELD	0.132	0.065	4.156	0.041	1.141 (1.005, 1.295)
SGK1	1.442	0.550	6.876	0.009	4.231 (1.439, 12.435)
Constant	-6.892	2.672	6.654	0.010	0.001

MELD, model for end stage liver disease; SGK1, serum/glucocorticoid-regulated kinase 1.

### 3.4 Performance of the MELD-S model in the development cohort

To verify the accuracy of the MELD-S model in predicting 90-day mortality risk in patients with ACLF, ROC curves were plotted, and Hosmer-Lemeshow test, DCA, and visualization were performed.

#### 3.4.1 ROC analysis and model fit

The MELD-S model yielded an AUC of 0.865 (95% CI: 0.767–0.963, *P* < 0.001), compared with the MELD score alone (AUC = 0.735, 95% CI: 0.594-0.876, *P* = 0.005) and SGK1 alone (AUC = 0.832, 95% CI: 0.719-0.944, *P* < 0.001). The *P* values reported with the individual AUC estimates represent tests against the null hypothesis of AUC = 0.5, not pairwise comparisons between MELD-S and the other models. Internal validation using 1, 000 bootstrap resamples yielded an optimism-adjusted AUC of 0.842, compared with the apparent development-cohort AUC of 0.865.

To evaluate the potential influence of propensity score matching on model performance, sensitivity analyses were conducted using the complete unmatched development cohort. When the final MELD-S equation was applied without refitting, MELD-S achieved an AUC of 0.842 (95% CI: 0.742–0.942) compared with 0.721 (95% CI: 0.594–0.848) for MELD alone. DeLong’s test showed that the difference between the two ROC curves remained significant (*P* = 0.041). At the optimal cutoff value of 0.40, the sensitivity was 88.0% and specificity was 66.7% (**Figure 2a**). The Hosmer–Lemeshow test indicated good model fit (χ² = 5.092, *P* = 0.748), with no significant difference between predicted and observed values.

**Figure 2:**
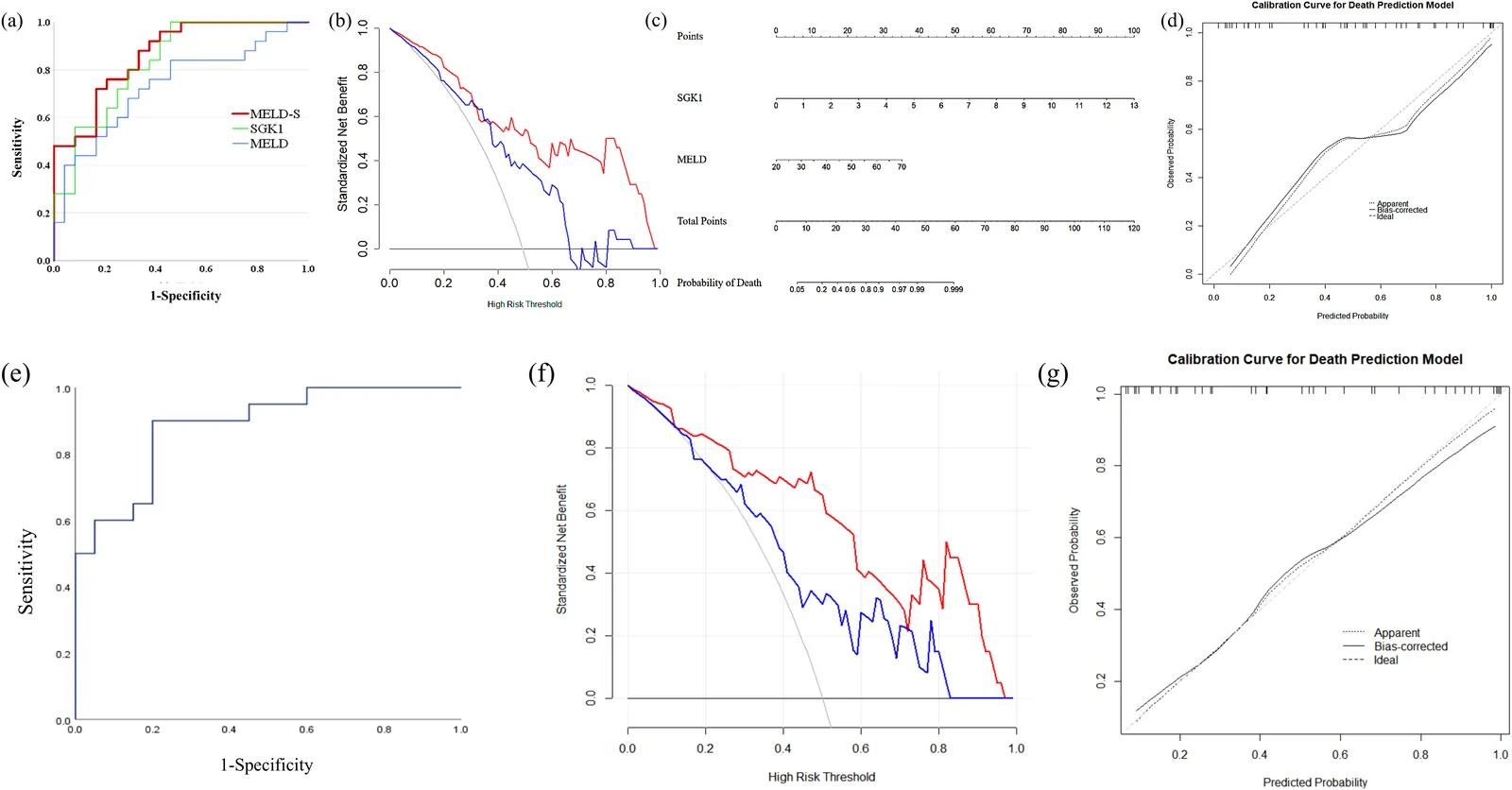
Development and external validation of the MELD-S prognostic model. **(a)** Receiver operating characteristic (ROC) curve analysis in the development cohort. The MELD-S model (red; AUC = 0.865) demonstrated superior discriminative ability for 90-day mortality compared to the MELD score (blue; AUC = 0.735) or SGK1 alone (green; AUC = 0.832). **(b)** Decision curve analysis (DCA) in the development cohort, showing the MELD-S model provided higher net clinical benefit than the MELD score across a range of threshold probabilities (0.3–0.8). **(c)** Nomogram for individualized prediction of 90-day mortality risk based on SGK1 level and MELD score. **(d)** Calibration curve in the development cohort, indicating good agreement between predicted and observed mortality risks (mean absolute error = 0.060). **(e)** ROC curve in the independent external validation cohort (AUC = 0.725), presented as a stepwise empirical curve, demonstrating maintained discriminative performance. **(f)** Calibration curve in the validation cohort, demonstrating satisfactory agreement between prediction and observation (mean absolute error = 0.26). **(g)** DCA in the validation cohort, where the MELD-S model (red) maintained a higher net benefit than the MELD score (blue) across clinically relevant risk thresholds.

#### 3.4.2 Calibration and decision curve analysis

The calibration curve demonstrated close alignment with the ideal line, with a mean absolute error (MAE) of 0.060, indicating high consistency between predicted probabilities and actual outcomes. DCA revealed that the MELD-S model (0.25-0.45) provided higher net benefit than the MELD score (0.15-0.30) across threshold probabilities of 0.3–0.8 (**Figure 2b**).

#### 3.4.3 Nomogram visualization

A nomogram was developed to visualize 90-day mortality risk for patients with ACLF based on SGK1 level and MELD score (**Figure 2c**). For example, a patient with SGK1 = 2.0 ng/mL and MELD score=35: SGK1 corresponds to approximately 15 points on the “Points” axis, MELD score corresponds to approximately 12 points, with a total score of approximately 27 points. The 90-day mortality probability corresponding to the total score on the “Probability of Death” axis is approximately 70%. The calibration curve for the nomogram also showed strong agreement with actual outcomes (MAE = 0.060) (**Figure 2d**).

#### 3.4.4 Comparison with established ACLF prognostic scores

To further evaluate the incremental prognostic value of MELD-S, its performance was compared with established ACLF prognostic models, including AARC, CLIF-C ACLF, and COSSH-ACLF scores, in the matched development cohort. The MELD-S model demonstrated the highest discriminative ability for predicting 90-day mortality (AUC = 0.865), compared with MELD (AUC = 0.735), AARC (AUC = 0.782), CLIF-C ACLF (AUC = 0.801), and COSSH-ACLF (AUC = 0.824). DeLong’s test showed that MELD-S had significantly better discrimination from MELD (*P* = 0.032) and AARC (*P* = 0.048), whereas the differences between MELD-S and CLIF-C ACLF (*P* = 0.071) or COSSH-ACLF (*P* = 0.146) were not statistically significant (**Table 3**).

**Table 3:** Comparison of prognostic performance among MELD-S and established ACLF scoring systems.

Model	AUC	95% CI	Sensitivity (%)	Specificity (%)	*P* value vs MELD-S (DeLong’s test)
MELD-S	0.865	0.767–0.963	88.0	66.7	Reference
MELD	0.735	0.594–0.876	72.0	68.0	0.032
AARC	0.782	0.651–0.913	76.0	72.0	0.048
CLIF-C ACLF	0.801	0.684–0.918	80.0	70.0	0.071
COSSH-ACLF	0.824	0.713–0.935	84.0	72.0	0.146

P values accompanying individual AUCs test the null hypothesis of AUC = 0.5. Pairwise P values are from DeLong’s test comparing each scoring system with MELD-S.

### 3.5 External validation of the MELD-S model

In the matched external validation cohort (n = 40), MELD-S yielded an AUC of 0.725 (95% CI: 0.563–0.887, *P* = 0.015; **Figure 2e**). In the complete unmatched validation cohort (n = 52), the fixed MELD-S equation yielded an AUC of 0.748 (95% CI: 0.620–0.876), compared with 0.691 (95% CI: 0.552–0.830) for MELD alone; the difference was not statistically significant by DeLong’s test (*P* = 0.182). The Hosmer–Lemeshow test yielded χ² = 3.166 and *P* = 0.924, and the calibration MAE was 0.26 (**Figure 2f**). Decision curve analysis for the validation cohort is presented in **Figure 2g**.

## 4 Discussion

By integrating transcriptomic screening with clinical evaluation, this study identified serum SGK1 as a candidate prognostic biomarker in ACLF. Higher serum SGK1 was associated with greater disease severity and independently associated with the 90-day outcome in the development cohort. Incorporating SGK1 into MELD improved discrimination in that cohort, and the resulting MELD-S model was subsequently evaluated in a separate validation cohort. Given the poor prognosis of ACLF (16), these findings support further evaluation of serum SGK1 as an additional source of prognostic information.

### 4.1 Unraveling the molecular nexus of regeneration failure in ACLF

Our multi-dataset bioinformatics approach identified 20 candidate genes that were associated with both liver failure (downregulated) and hepatocyte regeneration (upregulated), with enrichment in the FOXO1 signaling pathway. Among these, SGK1, GADD45β, and FOXO1 showed the highest connectivity in the PPI network and were selected as candidate regeneration-associated genes. Although enrichment analyses revealed their potential roles in mediating hepatocyte regeneration, these bioinformatics findings did not constitute clinical validation and should be considered hypothesis-generating, because the public transcriptomic datasets were small and differed in biological context (ACLF liver biopsies in GSE139602 and post-partial hepatectomy liver samples in GSE15239), microarray platform, sampling context, and data-processing history. In addition, the large number of DEGs identified in GSE139602 despite its small sample size warrants cautious interpretation. Therefore, the overlapping genes represent directionally concordant candidates rather than a validated molecular signature.

Subsequent clinical validation revealed a more complex and clinically relevant narrative than anticipated from bioinformatics alone: only SGK1 was independently associated with 90-day mortality, whereas GADD45β and FOXO1 did not remain independent predictors. This discrepancy may reflect differences between tissue-level transcription and circulating protein levels, and it underscores the need for further mechanistic validation. Despite these limitations, this integrated approach identified SGK1 as the only clinically validated independent predictor and supported the development of the MELD-S model, highlighting the value of combining bioinformatics discovery with prospective clinical validation.

#### 4.1.1 SGK1: a potential biomarker associated with disease severity in ACLF

SGK1, a serine/threonine kinase (17), is widely recognized for its pro-regenerative role and exerts a multifaceted function in coordinating hepatocellular regeneration, a function evidenced in liver repair models. Following partial hepatectomy, SGK1 has been shown to be essential for hepatocyte regeneration. Its knockdown markedly delayed the regenerative process by 48 hours, reduced the liver-to-body weight ratio by 28%, and curtailed the proportion of S-phase hepatocytes from 12% to 5%. This result is underpinned by a molecular mechanism whereby SGK1 directly phosphorylates ERK2 at Ser29, thereby stabilizing the MEK/ERK complex and amplifying a core pro-proliferative signal (18). In addition, SGK1 activation attenuates apoptosis and necrosis cascade by suppressing NF-κB signaling pathway (19) and confers anti-apoptotic protection through modulation of phosphatidylinositol-3 kinase pathway (20) and FOXO3a-Bim axis (6).Collectively, these experimental findings provide potential evidence linking SGK1 expression to the prognosis of liver failure.

The higher serum SGK1 concentrations in non-survivors, together with its positive correlation with MELD and independent association with the 90-day mortality, support the prognostic value of circulating SGK1 in this cohort. These clinical associations are compatible with the regenerative functions described in experimental models (21, 22), because circulating protein concentrations do not directly reflect tissue-specific signaling activity. Whether SGK1 exerts protective or detrimental effects during ACLF progression remains undetermined. Cytokine measurements, analyses linking SGK1 to inflammatory markers, and assessments of JNK-mediated SGK1 phosphorylation or NLRP3-inflammasome activation were not performed. These mechanisms therefore remain hypotheses for future investigation. Despite these limitations, the present findings identify SGK1 as an independent prognostic biomarker and provide a rationale for future mechanistic studies.

#### 4.1.2 GADD45β and FOXO1: candidate regeneration-associated proteins in ACLF

GADD45β and FOXO1 were also identified as candidate regeneration-associated genes through bioinformatic screening. GADD45β, a stress-responsive nuclear protein, facilitates DNA damage repair and promotes regenerative processes by recruiting transcription complexes to specific promoter regions and guiding its precise location (23). This functional role is further supported by a mouse model of partial hepatectomy, in which GADD45β knockout led to dysregulated stress-activated kinase signaling and significantly impaired hepatocyte proliferation, whereas its presence in wild-type mice suppressed excessive kinase activation, reduced apoptosis, and promoted hepatocyte survival and regeneration (24). Both SGK1 and GADD45β were significantly enriched in the FOXO1 signaling pathway, which serves as a central regulator of autophagy, proliferation, and apoptosis (8). Under physiological conditions, FOXO1 activation promotes autophagic flux and metabolic adaptation, essential for hepatocyte survival and regeneration (25). However, in the ACLF microenvironment, inflammatory mediators and oxidative stress induce hyperactivation of the PI3K/Akt pathway, leading to FOXO1 phosphorylation and its subsequent sequestration in the cytoplasm (9). This mislocalization abolishes FOXO1-mediated transcription of pro-autophagic and regenerative genes, thereby disrupting hepatocyte proliferation. Similar pathogenic FOXO1 activation has been implicated in M1 macrophage polarization and inflammation in non-alcoholic fatty liver disease (26), underscoring its potential role in liver failure.

The PPI network suggested close functional relationships among SGK1, GADD45β, and FOXO1 at the protein-interaction level. However, the bioinformatics analysis was based on liver-tissue transcriptomic datasets, whereas the clinical validation measured circulating serum protein concentrations by ELISA. Therefore, the higher serum concentrations observed in non-survivors should not be interpreted as evidence of tissue-level transcriptional upregulation or effective regeneration. Circulating protein levels may reflect systemic stress responses, disease severity, altered protein release or clearance, or compensatory responses, and may therefore differ from tissue-level mRNA expression. Importantly, only SGK1 was independently associated with 90-day mortality in the clinical cohort, whereas GADD45β and FOXO1 remain candidate proteins requiring further clinical and mechanistic validation. This integrated approach thus identifies SGK1 as a clinically validated prognostic biomarker and provides a focused direction for future mechanistic studies.

### 4.2 The MELD-S model: a biomarker-integrated approach for prognostic assessment

The principal contribution of MELD-S is the integration of serum molecular biomarker SGK1 with an established clinical severity score. In the development cohort, MELD-S achieved an AUC of 0.865 compared with 0.735 for MELD alone. Higher discrimination was also observed in the complete unmatched development cohort (AUC 0.842 versus 0.721; DeLong’s test *P* = 0.041), indicating that the improvement was not solely attributable to propensity score matching. In the complete unmatched validation cohort, MELD-S had a numerically higher AUC than MELD (0.748 versus 0.691), although the difference was not statistically significant (*P* = 0.182). Taken together, these results support the incremental prognostic value of adding SGK1 to MELD in the development cohort, while the magnitude and reproducibility of this improvement require confirmation in larger cohorts. Because MELD-S requires only one additional serum SGK1 measurement beyond the routinely used MELD score, it may offer a practical biomarker-assisted approach to risk assessment. Assay availability, cost, turnaround time, calibration, and clinical benefit should be evaluated prospectively.

### 4.3 Limitations and future perspectives

Our study has several limitations. First, the single-center design and modest sample size may affect the generalizability of our findings. Large-scale, multicenter prospective studies are therefore warranted to rigorously validate the MELD-S model across diverse etiologies and healthcare settings. Second, exclusion of patients with severe pre-existing cardiac, renal, or respiratory dysfunction may have introduced selection bias and may limit the applicability of the model to these populations. Third, propensity score matching was applied during model development to reduce baseline imbalance. However, outcome-based matching may alter the original clinical spectrum and influence discrimination estimates. Although sensitivity analyses using unmatched cohorts supported the incremental value of MELD-S, larger prospective studies using consecutive patients without outcome-based matching are required for definitive assessment of model performance. Fourth, the lower AUC in the external validation cohort indicates that development performance may not be fully reproduced in subsequent patients, highlighting the need for validation in larger, consecutively enrolled cohorts. Fifth, although bootstrap internal validation suggested relatively stable performance, multicenter validation and calibration assessment remain necessary. In addition, our hypothesis-driven focus on regeneration-associated genes identified SGK1, but the complex pathophysiology of ACLF involves numerous other pathways. Future research should explore the integration of SGK1 with other dynamic biomarkers, such as inflammatory cytokines (e.g., IL-6) or regenerative markers (e.g., alpha-fetoprotein), to construct more robust multimodal prediction panels. The mechanisms linking circulating SGK1 to ACLF severity and mortality, including any interactions with stress- or inflammasome-related signaling, remain to be determined experimentally, and experimental studies are needed before therapeutic implications can be considered (27). Moreover, outcomes in ACLF can be influenced by individual treatment adherence, and follow-up compliance, which may affect the clinical outcomes and enrollment, representing an additional potential source of variability.

Notwithstanding these limitations, this study provides a proof of concept for integrating a molecular biomarker with an established clinical score in ACLF. By identifying SGK1 as an independent prognostic biomarker and developing the MELD-S model, our findings offer a practical framework for biomarker-assisted risk stratification and provide a clear rationale for future multicenter validation and mechanistic investigation.

## 5 Conclusion

In conclusion, by integrating bioinformatics discovery with prospective clinical validation, this study identified SGK1 as a novel independent predictor of 90-day mortality in ACLF. The derived MELD-S model, which incorporates SGK1 with the established MELD score, showed improved prognostic performance and offers a practical biomarker-assisted approach for risk stratification in ACLF. Although bioinformatics analyses suggest that SGK1 may be involved in regeneration-related signaling pathways, the biological mechanisms underlying its association with ACLF outcomes require further experimental validation. Future multicenter prospective studies are warranted to validate the reproducibility, calibration, and clinical applicability of the MELD-S model across diverse ACLF populations. This integrated approach provides a proof of concept for combining molecular biomarkers with clinical scores to improve prognostic assessment in ACLF.

## Data Availability

The datasets presented in this study can be found in online repositories. The names of the repository/repositories and accession number(s) can be found in the article/**Supplementary Material**.
