# Supplementary material for: MELD-S prognostic model: development and external validation for 90-day mortality prediction in acute-on-chronic liver failure
Source: Front Immunol. 2026 Oct 21;17:1881613. doi: 10.3389/fimmu.2026.1881613 (PMC13731366; doi:10.3389/fimmu.2026.1881613)
Supplement: Supplementary file 1 [file Table1.docx]

**Supplementary Tables**

**Supplementary Table 1** Comparison of baseline characteristics between survivor and non-survivor groups in the development cohort

| **Variable** | **Survivor group**  **(n = 25)** | **Non-survivor group**  **(n = 25)** | **Statistics**  **(t/*χ*²)** | ***P* value** |
| --- | --- | --- | --- | --- |
| **Gender (male, %)** | 16 (64) | 14 (56) | 0.333 | 0.564 |
| **Age (year)** | 53±13 | 52±9 | 0.206 | 0.838 |
| **Smoking history (n, %)** | 11 (44) | 11 (44) | 0 | 1.000 |
| **Alcohol history (n, %)** | 15 (60) | 12 (48) | 0.725 | 0.395 |
| **Etiology of liver disease** |  |  |  |  |
| Virus, n (%) | 4 (16) | 11 (44) | 7.111 | 0.130 |
| Non-virus, n (%) |  |  |  |  |
| Alcohol | 8 (32) | 7 (28) |  |  |
| Drugs | 5 (20) | 5 (20) |  |  |
| Others | 7 (28) | 2 (8) |  |  |
| **Comorbidity** |  |  |  |  |
| Diabetes n (%) | 3 (12) | 3 (12) | 0 | 1.000 |
| Hypertension n (%) | 1 (4) | 6 (24) | 4.153 | 0.042 |
| **Complications at baseline** |  |  |  |  |
| HE n (%) | 2 (8) | 2 (8) | 0 | 1.000 |
| Infection n (%) |  |  |  |  |
| Pulmonary infection | 5 (20) | 6 (24) | 5.209 | 0.267 |
| Abdominal infection | 2(8) | 0 |  |  |
| Ascites n (%) | 6 (24) | 10 (40) | 1.471 | 0.225 |
| Renal dysfunction n (%) | 2 (8) | 3 (12) | 0.222 | 0.637 |

Note: HE, hepatic encephalopathy

**Supplementary Table 2** Comparison of baseline characteristics between survivor and non-survivor groups in the external validation cohort

| **Variable** | **Survivor group**  **(n = 20)** | **Non-survivor group**  **(n = 20)** | **Statistics**  **(t/*χ*²)** | **P value** |
| --- | --- | --- | --- | --- |
| **Gender (male, %)** | 14 (70) | 17 (85) | -1.290 | 0.256 |
| **Age (year)** | 50 ± 9 | 49 ± 12 | 0.206 | 0.838 |
| **Smoking history (n, %)** | 8 (40) | 9 (45) | 0.102 | 0.749 |
| **Alcohol history (n, %)** | 11 (55) | 10 (50) | 0.100 | 0.752 |
| **Etiology of liver disease** |  |  |  |  |
| Virus, n (%) | 6 (30) | 9 (45) | 1.761 | 0.413 |
| **Comorbidity** |  |  |  |  |
| Diabetes n (%) | 3 (15) | 3 (15) | 0 | 1.000 |
| Hypertension n (%) | 2 (10) | 4 (20) | 0.444 | 0.654 |
| **Complications at baseline** | |  |  |  |
| HE n (%) | 8(40) | 13(65) | 2.506 | 0.113 |
| Infection n (%) | 12(60) | 16(80) | 1.905 | 0.168 |
| Ascites n (%) | 13(65) | 15(75) | 0.476 | 0.490 |
| Renal dysfunction n (%) | 1 (5) | 3 (15) | 0.297 | 0.607 |

Note: HE, hepatic encephalopathy

**Supplementary Table 3** Comparison of laboratory parameters between survivor and non-survivor groups in the development cohort

| **Variable** | **Survivor group**  **(n = 25)** | **Non-survivor group**  **(n = 25)** | **Statistics**  **(Z/t)** | ***P* value** |
| --- | --- | --- | --- | --- |
| WBC (×10^9^/L) | 5.62 (4.90,11.41) | 6.58 (4.39,13.47) | 0.087 | 0.930 |
| NEUT (×10^9^/L) | 3.49 (2.76,8.68) | 5.39 (2.59,11.52) | 0.243 | 0.808 |
| LYM (×10^9^/L) | 1.34(0.66,1.94) | 0.96(0.71,1.37) | -1.028 | 0.304 |
| RBC (×10^9^/L) | 2.99±0.94 | 2.79±0.79 | -0.797 | 0.429 |
| PLT (×10^9^/L) | 112.00(69.00,170.00) | 92(42.50,140.00) | -0.873 | 0.383 |
| PT (s) | 20.90(17.35,24.35) | 29.20(21.95,35.90) | 2.872 | 0.004 |
| PTA (%) | 41.00(34.50,52.50) | 28(21.5,39.00) | -2.873 | 0.004 |
| INR | 1.87(1.56,2.18) | 2.61(1.96,3.20) | 2.872 | 0.004 |
| APTT (s) | 38.30(34.35,43.10) | 46.7(38.15,56.10) | 2.571 | 0.010 |
| Fib (g/L) | 1.52(1.11,1.88) | 1.24(0.99,1.93) | -0.786 | 0.432 |
| D-dimer (μg/mL) | 2.05(1.28,5.43) | 4.62(2.87,6.70) | 2.054 | 0.040 |
| ALB (g/L) | 30.00(27.65,32.05) | 30.6(27.10,32.40) | 0.039 | 0.969 |
| ALT (U/L) | 50.00(21.50,245.50) | 66(27.00,125.50) | 0.126 | 0.900 |
| AST (U/L) | 112.00(51.50,286.00) | 79.00(42.00,160.50) | -1.009 | 0.313 |
| TBiL (μmol/L) | 247.96±97.77 | 303.80±112.27 | 1.876 | 0.067 |
| ALP (U/L) | 148.00(97.50,177.00) | 126(95.00,195.00) | -0.301 | 0.764 |
| GGT (U/L) | 90.00(42.50,212.00) | 57(34.50,197.30) | -1.087 | 0.277 |
| Na^+^ (mmol/L) | 134.60±4.16 | 135.88±7.95 | 0.713 | 0.479 |
| SCr (umol/L) | 62.00(45.00,76.00) | 68(50.50,114.50) | 1.271 | 0.204 |
| MELD | 32.39(30.22,37.76) | 39.00(34.93,44.38) | 2.978 | 0.003 |
| MELD-Na | 34.10±8.74 | 39.39±13.33 | 0.797 | 0.122 |
| AFP (μg/L) | 6.80 (4.00,104) | 6.40 (3.77,18.15) | 0.877 | 0.380 |
| SGK1 (ng/mL) | 0.41(0.15,1.67) | 1.99(1.53,7.63) | 3.980 | <0.001 |
| GADD45β(ng/mL) | 0.89(0.57,1.11) | 1.09(0.95,7.96) | 3.150 | 0.002 |
| FOXO1 (ng/mL) | 1.32 (0.89,1.66) | 1.70(1.53,8.98) | 3.469 | 0.001 |

Note: WBC, white blood cell; NEUT, neutrophilic granulocyte; LYM, lymphocyte; RBC, red blood cell; PLT, platelets; PT, prothrombin time; PTA, prothrombin activity; INR, international normalized ratio; APTT, activated partial thromboplastin time; Fib, fibrinogen; ALB, albumin; ALT, alanine aminotransferase; AST, aspartate aminotransferase; TBil, total bilirubin; ALP, alkaline phosphatase; GGT, gamma-glutamyl transpeptidase; Na, sodion; SCr, serum creatinine; MELD, model for end stage liver disease; AFP, alpha-fetoprotein; SGK1, serum/glucocorticoid-regulated kinase 1; GADD45β, growth arrest and DNA-damage-inducible protein 45 beta; FOXO1, forkhead box protein O1

**Supplementary Table 4** Comparison of laboratory data between survivor and non-survivor groups in the external validation cohort

| **Variable** | **Survivor group**  **(n = 20)** | **Non-survivor group**  **(n = 20)** | **Statistics**  **(Z/t)** | ***P* value** |
| --- | --- | --- | --- | --- |
| WBC (×10^9/^L) | 5.86 (4.2, 11.3) | 7.12 (5.6, 11.1) | -1.136 | 0.256 |
| NEUT (×10^9^/L) | 5.32 (2.7, 9.6) | 5.55 (3.7, 8.8) | -0.460 | 0.646 |
| LYM (×10^9^/L) | 1.07 (0.7, 1.8) | 0.98 (0.7, 1.3) | 0.352 | 0.725 |
| RBC (×10^9^/L) | 3.04 (2.3, 3.8) | 3.57 (2.8, 4.3) | -1.256 | 0.209 |
| PLT (×10^9^/L) | 73.50 (48.8, 127.8) | 72.00 (52.3, 106.0) | 0.338 | 0.735 |
| PT(s) | 25.70 (19.3, 31.9) | 24.40 (20.2, 34.0) | 0.590 | 0.555 |
| PTA (%) | 37.00 (26.5, 47.5) | 30.00 (17.3, 40.5) | 1.692 | 0.091 |
| INR | 2.06 (1.7, 2.7) | 2.65 (2.0, 3.9) | -2.015 | 0.044 |
| APTT(s) | 41.70 (36.6, 47.6) | 48.05 (37.6, 59.3) | -1.515 | 0.130 |
| Fib (g/L) | 1.56 (1.2, 1.7) | 1.36 (0.8, 1.6) | 1.154 | 0.248 |
| ALB (g/L) | 28.65 (27.1, 32.5) | 30.55 (27.2, 33.4) | -0.541 | 0.588 |
| ALT (U/L) | 38.00 (22.7, 150.5) | 61.00 (31.0, 379.5) | -1.285 | 0.199 |
| AST (U/L) | 81.00 (46.8, 280.3) | 105.50 (75.5, 295.8) | -1.258 | 0.208 |
| TBiL (μmol/L) | 282.65 (66.7, 349.0) | 296.70 (197.5, 441.8) | -1.325 | 0.185 |
| ALP (U/L) | 124.00 (97.8, 171.5) | 120.00 (100.3, 188.0) | 0.162 | 0.871 |
| GGT (U/L) | 46.50 (22.3, 199.3) | 67.50 (46.0, 127.3) | -0.839 | 0.402 |
| Na^+^ | 133.00 (127.0, 135.0) | 135.00 (129.0, 137.0) | -0.786 | 0.432 |
| Cr (umol/L) | 64.50 (51.0, 77.4) | 58.50 (50.0, 74.0) | 0.447 | 0.655 |
| MELD | 18.48 (13.1, 24.4) | 24.39 (19.6, 29.7) | -2.570 | 0.010 |
| MELD-Na | 24.86 (13.1, 32.1) | 24.88 (19.1, 35.1) | -0.520 | 0.603 |
| SGK1 (ng/mL) | 0.80 (0.4, 1.1) | 1.19 (1.0, 5.7) | -3.381 | 0.001 |

Note: WBC, white blood cell; NEUT, neutrophilic granulocyte; LYM, lymphocyte; RBC, red blood cell; PLT, platelets; PT, prothrombin time; PTA, prothrombin activity; INR, international normalized ratio; APTT, activated partial thromboplastin time; Fib, fibrinogen; ALB, albumin; ALT, alanine aminotransferase; AST, aspartate aminotransferase; TBil, total bilirubin; ALP, alkaline phosphatase; GGT, gamma-glutamyl transpeptidase; Na, sodion; SCr, serum creatinine; MELD, model for end stage liver disease; SGK1, serum/glucocorticoid-regulated kinase 1.
